# Legal Murder

**Published:** 1893-04

**Authors:** 


### EDITORIAL DEPARTMENT.

F. E. DANIEL, M. D., Editor.
S. E. HUDSON, M. D., Managing Editor.
A. J. SMITH. M. D., Galveston, Associate Editor.

EDITORIAL STAFF:

PROF. J. E. THOMPSON, M. D., Texas Medical College, Galveston; Surgery.
PROF. WM. KEILLER, M. D., Texas Medical College, Galveston; Obstetrics and Gynecology.
PROF. DAVID CERNA, M. D., Texas Medical College, Galveston; Therapeutics.
PROF. A. J. SMITH, M. D., Texas Medical College, Galveston; Medicine.
DR. ISADORE DYER, Tulane University; Dermatology.
DR. R. H. L. BIBB, Saltillo, Mexico; Foreign Correspondent.
DR. ROBT. MORRIS, Charity Hospital, N. O.; Clinical Reports.

———o———

The JOURNAL is the official organ of the Austin District Medical Society, the Wes Texas District Medical Society and the Galveston County Medical Society.

### LEGAL MURDER

The legal .infliction of death, under sentence of the court, is a horrible thing to comtemplate under any circumstances, and humanity revolts at it. It is a sin in the sight of heaven, and should be abolished; it is a stain upon our civilization.

Murder is a great crime. We have never been able to understand how, by murdering the murderer, even with' the sanction and the authority of the law, it can be rendered less a crime, or, in any way, repair the consequences of the first murder. It is to be condemned from every standpoint. Its only excuse is a lame one; it is ostensibly, to strike such awe in the minds of the evil disposed as to deter them from crime. But statistics show that in States where capital punishment is the law, crime is actually on the increase. We believe it can be shown that it suggests crime in the minds of those who witness it; thus, it fails of its only pretext, its sole *raison d'etre.* It is to be condemned on the score of economy; for every adult person is said to be worth one thousand dollars a year as a producer.

But, a man, by the commission of a great crime, forfeits his right to live in a civilized community, they say. That is no reason why he should be put to death. Separate him from the world, and put him to work for the benefit of those whose society he has forfeited.

But, so long as murder by a sheriff is made legal, in the name of humanity, let it be inflicted in a manner the least horrible and revolting.

New York State substituted electro-cution, as death by electricity is commonly called, for hanging by the neck, as being, theoretically, less brutal and revolting; but in this respect it has proved a failure. *Nothing* could be more harrowing and disgusting to a Christian man or woman than were the accounts of the earlier electro-cutions at Sing-sing. We have just read the account of a recent execution there which is some improvement on the first (they are getting their hands in, we suppose); and this account has suggested that, so long as a man is to suffer death for his crime, let some method be adopted that will accomplish the end rapidly, surely, and without pain to the criminal, or disgust to the spectators or perpetrators; and it occurs to us that that end can be secured by a hypodermic injection *of a drachm or two of hydrocyanic acid!* Accounts of suicide by swallowing Prussic acid all agree that death is nearly instantaneous, and, it is to be reasonably inferred, painless; the suicide has not time to drop the vial, or take it from his lips.

We believe this a good suggestion. It is made in the name of humanity, and we would like to see it seriously considered, and discussed by the medical press. It would be interesting to ascertain the smallest amount of the poison which would, if given hypodermically, be equal to the thousands of "volts" it requires to murder a man; we have no statistics on hand on this point, but it is known that a very small quantity will kill if swallowed. Oily Gammon, and Jonas Chuzzlewit, are supposed to have cheated the gallows by the use of not over a drachm or so; It is known that many medicines are more active when hypodermically given than by the mouth, and it is fair to assume that a very small quantity of this acid would be sufficient to murder a convicted criminal. Let this method then be given a trial, at least; it certainly can be no worse than hanging,—ugh—or, the so called "painless death" by electro-cution.

